# Validation of the scale for assessing the psychological vulnerability and its association with health of intimate partner violence victims in Chinese young adult population

**DOI:** 10.1371/journal.pone.0235761

**Published:** 2020-07-06

**Authors:** Anna W. M. Choi

**Affiliations:** Department of Social and Behavioural Sciences, City University of Hong Kong, Hong Kong, Hong Kong; Universidad Pública de Navarra, SPAIN

## Abstract

The Women’s Experience with Battering (WEB) scale is a self-report instrument that uses a 10-item Likert-type scale to measure IPV victims’ cognitive and affective experience of battering. This study aimed to validate the Chinese version of the WEB scale using gender-neutral questions, Experience of Battering Scale (Chinese) (EBS-C), to assess the psychological vulnerability of victims of intimate partner violence (IPV). The study adopted a range of methods, including translation and back translation, expert reviews, cognitive debriefing, and test-retest reliability assessment. The EBS-C was validated in a purposive convenience sample of 718 Chinese-speaking participants (male = 362; female = 356) aged 18–24 (mean age = 21.4) in Hong Kong. The results of CFA showed a good model fit: CFI = .97, TLI = .96, RMSEA = .05, SRMR = .03. The EBS-C was also found to be significantly associated with the Revised Conflict Tactics Scales (CTS2; r = .13–.17, p < .01), depression (BDI-II: r = .15, p < .01), anxiety (GAD-7: r = .17, p < .01), interpersonal support (ISEL-12: r = -.27, p < .01), relationship satisfaction (RAS: r = -.36, p < .01), and self-esteem (RSES: r = -.22, p < .01). The study demonstrated the EBS-C to be a reliable and valid measure for assessing the psychological vulnerability of IPV victims. It is thus useful for identifying the risks such individuals face by assessing their experience of fear, danger, and disempowerment in the intimate relationship relative to abusive incident-based measures alone. The EBS-C will also be useful for developing effective treatments to address the psychological vulnerability resulting from IPV and will facilitate cross-cultural comparative research aimed at enriching our knowledge of IPV victimization.

## Introduction

Intimate partner violence (IPV) is a worldwide public health concern that affects the physical and mental health of both victims and their children [1]. Many scales have been developed to measure the occurrence and severity of IPV. Most such assessment/screening tools, including the popular incidence-based Revised Conflict Tactics Scales (CTS2) [2] and Abuse Assessment Screen (AAS) [3], quantify the frequency of physical, sexual, and psychological abuse in the intimate relationship, but fail to capture the cognitive and affective experience of battery for the women involved, disempowerment and fear in particular. The Women’s Experience with Battering (WEB) scale was designed to gain a better understanding of women’s psychological vulnerability arising from being battered, which is useful for developing effective prevention and intervention strategies [4]. With IPV being a worldwide public health concern, it is essential to translate the scale into other languages. The study reported herein was thus conducted to validate the Chinese version of the WEB scale using gender-neutral questions, Experience of Battering Scale-Chinese (EBS-C), to assess the needs of Chinese families and facilitate cross-cultural studies of IPV.

### Common incidence-based screening and assessment tools

The CTS2 is a common IPV assessment tool used for both men and women. It comprises 78 self-report items representing the IPV-related behaviors of respondents and their intimate partners, and measures IPV severity by the frequency of instances of negotiation, physical assault, sexual coercion, injury, and psychological aggression [5]. Similar to the CTS2 is the Index of Spouse Abuse (ISA), which measures the severity of IPV by the frequency with which IPV-related behaviors occur [6]. However, ISA perceives women as the victims of IPV, and thus targets only female respondents. It also contains only 30 self-report items, far fewer than the CTS2. It can be an effective tool in time-sensitive settings; however, with Hudson and McIntosh [6] showing that it can be completed within 5 minutes by most respondents.

The CTS2 emphasizes measurement of the frequency of IPV-related behaviors, whereas the AAS explores whether women have ever suffered physical and psychological abuse, as well as sexual coercion [3]. The latter is regarded as an effective IPV screening scale that also evaluates female victims’ situation during pregnancy thanks to its five yes/no dichotomous items pertaining to IPV during the respondent’s lifetime, within the past year, and during pregnancy and three follow-up questions [7]. It is also a quicker IPV screening instrument for women than the CTS2 [8].

Similar to the AAS, the Dangerousness Assessment Scale (DAS) also measures the risks faced by female victims using yes/no dichotomous questions concerning different types of abuse [9]. It also collects details of the IPV experience by recording the dates of IPV occurrences in the past year and classifies them into five levels of physical abuse to identify an escalation in violence [9]. However, Lin and Sheng [10] have called the effectiveness of the DAS into question, arguing that many victims are unable to remember the exact dates of instances of physical abuse.

Tolman [11] developed the Psychological Maltreatment of Women Inventory (PMWI), whose 58 items were derived from ISA and the CTS-2 and based on his clinical observations. It explores the level of IPV that respondents have suffered by the frequency with which their partners engage in psychologically abusive behaviors. Although the PMWI focuses on just one type of IPV, psychological maltreatment is useful for in-depth exploration of female victims’ emotional state [11]. Many other IPV screening and assessment scales, including ISA, AAS, and DAS, also target female victims because women are the major victims of IPV globally [12].

### Women’s experience with Battering Scale

Different from incidence-based tools for assessing IPV, the WEB scale [13] is a 10-item Likert-type scale designed to address battered women’s perspectives on the meaning of battery or, in other words, their cognitive and affective experience of being battered. The theoretical foundation of the scale is focus group analysis of 22 women’s subjective experiences of physical and psychological danger and a loss of power and control in their relationship with a heterosexual partner [4]. Its ten items, which are rated on a 6-point Likert-type scale, reflect a single factor, namely, the underlying meaning of domestic violence for battered women. The WEB scale allows researchers to assess the intensity of a battered woman’s experience in terms of psychological vulnerability without relying on their arbitrary interpretation [13]. Prior studies have demonstrated the scale to exhibit good construct validity, with respondents receiving a total score higher than 19 regarded as battered [14, 15].

The WEB scale has also been modified to gender-neutral questions, e.g., my partner rather than she, to capture the perceived dating experiences of both male and female youths [16, 17] and perceptions of the intimate relationship among adults of both sexes [18–20], allowing the assessment of IPV experiences, the psychological vulnerability in particular, of both women and men.

### Chinese-language screening and assessment tools

A number of studies have been conducted in Chinese communities, including Hong Kong, mainland China, and Taiwan, to validate various scales for assessing and screening IPV, including the CTS2 [21, 22], ISA [23], Personal and Relationships Profile (PRP) [24], AAS [25, 26], DAS [27, 28], and PMWI [29].

In addition, Wang [30] developed the Taiwan Intimate Partner Violence Danger Assessment (TIPVDA) as an assessment tool for use in quantitative and qualitative analysis of Chinese samples. One part of the two-part instrument includes 15 yes/no dichotomous questions indicating the risk factors of IPV and self-perceived levels of the dangerousness of corresponding situations ranked on a scale ranging from 0 to 10, whereas the other part comprises interview notes on the important signals displayed by and information gleaned from the respondents during observations. The higher the score recorded from “yes” responses to the 15 dichotomous items, the greater the IPV risk faced by the respondent. Using a checklist comprising various types of life-threatening behavior perpetrated by intimate partners in the past or present, Wang [30] showed TIPVDA scores to be strongly correlated with the number of IPV episodes, with its mean score significantly higher when such behavior occurred in both the past and present, and significantly lower when it had never occurred at all. The implication is that the degree of danger depends to a significant extent on the number of episodes involving IPV-related behaviors.

A similar scale assessing the level of IPV risk was developed in Hong Kong, namely, the Brief Spousal Assault Form for the Evaluation of Risk (B-SAFER) [31]. The scale’s ten items indicating the risk factors for IPV are scored by the frequency with which those factors occur. The scale targets male offenders and explores their history of IPV and their functioning in terms of their emotional state and social life [31]. Au and colleagues [31] adopted the scale to assess two groups: one comprising men who had exhibited IPV-related behaviors in the past year (i.e., the batterer group) and the other comprising men who had never engaged in such behaviors (i.e., the comparison group). The mean scores of the batterer group were consistently higher than those of the comparison group. Their participants also completed the CTS2, with the B-SAFER scores found to be positively correlated with the psychological aggression and physical assault subscales of the CTS2 [31].

All of these Chinese-language assessment scales are incidence-based tools for measuring IPV. To date, no measurement tool in Chinese has been devised to assess the subjective experience of psychological vulnerability in male and female victims of IPV and the perpetrators of such violence. Psychological vulnerability in the IPV context is defined as the continual perception of susceptibility to physical and psychological danger, powerlessness, and a loss of control in one’s relationship with an intimate partner [13], and is considered an important factor in understanding the risks faced by IPV victims. The victimization is not only consisted of acts of violence but also a psychological vulnerability of susceptibility to psychological and/or physical danger, loss of power and control in a relationship [13]. Thus, a person’s psychological vulnerability can reflect his/her level of victimization under the abuse of power and control framework of IPV. In addition, psychological IPV often co-occurs with physical IPV [32] and victimization is highly associated to victims’ mental health problem, such as depression and post-traumatic stress disorder (PTSD) [33–35]. Therefore, the assessment of psychological vulnerability to identifying the level of victimization is important in measurement of IPV. The WEB scale incorporates psychological vulnerability but requires cross-cultural translation for use in Chinese populations. Furthermore, before the Chinese version of the scale, i.e., EBS-C, is ready for larger-scale psychometric evaluation, cognitive debriefing is needed to assess its relevance, clarity, and acceptability.

Accordingly, the aim of the study reported herein was to assess the translated traditional Chinese version of the WEB scale to evaluate its relevance, clarity, and acceptability. As the WEB scale has been modified to incorporate gender-neutral questions to measure the abuse of power, control, and fear in intimate relationships from the perspective of both female and male victims and perpetrators [19], gender-neutral pronouns, e.g., my partner, were used in the Experience of Battering Scale (Chinese) (EBS-C) examined in this study. The study’s findings will help researchers and practitioners alike to better understand the psychological vulnerability of both female and male victims and perpetrators of IPV in the Chinese context and facilitate the development of optimal interventions and preventive measurements for cases of IPV in Chinese populations.

## Method

### Participants

Participants for the subsequent validation study were recruited from tertiary institutions, youth centers, and employment training centers in Hong Kong. The inclusion criteria were Chinese ethnicity, aged 18–24, and current or past involvement in an intimate relationship. Those unable to read and understand Chinese and/or provide informed consent were excluded. Written consent was obtained from all participants prior to study commencement, and they were provided with an information sheet explicating the purpose, procedure, and potential benefits and risks of the study and an assurance that their personal data would remain confidential and anonymous. An information sheet of social services for helping individuals and families experienced domestic violence was also provided for the participants. The study was approved by the Institutional Review Board of The University of Hong Kong/Hospital Authority Hong Kong West Cluster.

### Adapting the WEB

#### Translation and back translation

After obtaining permission from the author of the original English-language version of the WEB scale [4], it was translated into the 10-item EBS-C and then back-translated into English by another translator without any knowledge of the English version. The two translators then reviewed their translated versions and modified them until the consensus version was comparable to the original.

#### Content validity judged by experts

The EBS-C was independently assessed and reviewed by an expert panel comprising two of the researchers, a social worker, and a nurse with expertise in conducting IPV research or providing community services to abused women. The panel was instructed to review the scale’s wording from three key perspectives: semantic equivalence (vocabulary and grammatic considerations), idiomatic equivalence (unfamiliar expressions or slang), and experience equivalence (“daily life” concept validity) [36]. To assess the EBS-C’s content validity, the experts were asked to rate its items in terms of the clarity (1 = *item is unclear*, 2 = *item needs major revisions to be clear*, 3 = *item needs minor revisions to be clear*, and 4 = *item is clear*) [37] and relevance (1 = *not relevant*, 2 = *unable to assess relevance without item revision*, 3 = *relevant but needs minor alteration*, 4 = *very relevant*) [38] of the underlying construct on a 4-point Likert scale. An item-level content validity index (I-CVI) was computed by the number of experts judging an item to be relevant or clear (rating of 3 or 4), divided by the total number of experts, and a scale-level content validity index (S-CVI) by the proportion of items achieving a rating of 3 or 4 [39]. The scores for both the I-CVI and S-CVI were higher than 80%, indicating that the items were considered relevant by most of the experts [40].

#### Cognitive debriefing

Ten Chinese men and women aged 18 or above were recruited for cognitive debriefing in August 2016 from tertiary institutions, youth centers, and employment training centers. The inclusion criteria were Chinese ethnicity, aged 18–65, and current or past involvement in an intimate relationship. Those unable to read and understand Chinese and/or provide informed consent were excluded. The mean age was 37.8 years (SD = 14.9) covering different socio-economic backgrounds. The recruited respondents completed the EBS-C and then took part in face-to-face interviews conducted in a research center to collect their responses to the items to identify any errors, difficulties, or confusing elements. A total of four items of the scale were then modified and refined based on these qualitative interview data to improve its comprehensibility and ensure its administration feasibility. Prior to the instrument’s validation, the revised version was further reviewed by the experts who assessed its content validity.

#### Test-retest reliability

718 Chinese participants were recruited to participate in the validation study and they were instructed to fill in a self-administered questionnaire. After a 1-week interval, they were asked to complete the EBS-C a second time. With recruitment at the community randomly, the sample was considered as a representative sample of a larger population. The scale’s intraclass correlation coefficient was .872, indicating good test-retest reliability [41].

### Measures

#### Experience of Battering Scale (Chinese) (EBS-C)

The original Women’s experience with battering (WEB) scale is a 10-item 6-point Likert-type scale (1 = *strongly agree*; 6 = *strongly disagree*) designed to assess psychological vulnerability [13]. As noted, it was later modified to gender-neutral questions to measure the abuse of power, control, and fear in the context of an intimate relationship in both female and male participants [19]. All ten items are reverse-coded, and the score range is from 10 to 60, with higher scores indicating a higher level of psychological vulnerability. The WEB scale has demonstrated good construct validity and the ability to accurately distinguish battered from non-battered women [4, 13, 42, 43]. It has also demonstrated strong internal consistency reliability (Cronbach’s alpha [α] = 0.91) [44]. Use of the cutoff point of > 19 to classify a woman as being battered resulted in a sensitivity of 94.6% and specificity of 96.1% [13].

#### Revised Conflict Tactics Scales (CTS2)

The CTS2 is used to assess the occurrence of interpersonal violence, including physical assault, injury, psychological aggression, and sexual abuse during the previous 12 months [5]. Examples of questions in CTS2 include “insulted or swore at partner” (psychological abuse), “pushed or shoved partner” (physical assault), “I had a sprain, bruise, or small cut because of a fight with the partner” (injury), “my partner used threats to make me have sex” (sexual abuse). The CTS2 has been translated into Chinese, with the Chinese version demonstrating satisfactory validating and reliability (α ranging from 0.79 to 0.95) [21].

#### Beck Depression Inventory version II (BDI-II)

The 21 item BDI-II is a self-report instrument used to assess symptoms corresponding to the criteria for diagnosing depressive disorders [45]. Examples of questions include Sadness–“I do not feel sad.”/“I feel sad much of the time”/“I am sad all the time.”/“I am so said or unhappy that I can’t stand it”; Past Failure–“I do not feel like a failure.”/“I have failed more than I should have.”/As I look back, I see a lot of failures.”/“I feel I am a total failure as a person.”. The BDI-II has been translated into Chinese, with the Chinese version demonstrating satisfactory validity and reliability (α ranging from .86 to .87) [46]. Higher scores indicate more depressive symptoms.

#### Generalized Anxiety Disorder-7 (GAD-7)

The 7-item GAD-7 is a widely used tool for assessing respondents’ anxiety symptoms over the past two weeks [47]. Examples of questions include “Feeling nervous, anxious or on edge”, “Trouble relaxing”, “Becoming easily annoyed or irritable”. The Chinese version has an internal consistency score of 0.88 [48]. Higher scores on the GAD-7 indicate a more severe generalized anxiety disorder.

#### Interpersonal Support Evaluation List-12 (ISEL-12)

The 12-item ISEL-12 is used to measure young adults’ perceived social support. Its three subscales cover appraisal (e.g., “I feel that there is no one I can share my most private worries and fears with.”), belonging (e.g., “If I decide one afternoon that I would like to go to a movie that evening, I could easily find someone to go with me.”), and tangible support (e.g., “If I were sick, I could easily find someone to help me with my daily chores.”). Each has four items whose scores range from 0 (definitely false) to 3 (definitely true), giving a total score ranging from 0 to 36. The higher the score, the greater the amount of social support that respondents perceive. The ISEL-12 has demonstrated good psychometric properties and good internal consistency (α = 0.88) [49].

#### Relationship Assessment Scale (RAS)

The 7-item RAS, which has good psychometric properties and good internal consistency (α = 0.86), is used to measure perceived relationship satisfaction in young adults. Examples of questions including “How well does your partner meet your needs?”, “In general, how satisfied are you with your relationship?”, “How much do you love your partner?”. Its item scores range from 1 (low satisfaction) to 5 (high satisfaction), giving a total score ranging from 7 to 35. The higher the score, the more satisfied respondents are with their romantic relationship [50].

#### Rosenberg Self-Esteem Scale (RSES)

The 10-item RSES is used to measure self-esteem, with higher scores indicating higher levels of self-esteem [51] and good internal consistency (α = 0.81). Examples of questions include “On the whole, I am satisfied with myself.”, “At times I think I am no good at all.”, “I feel I do not have much to be proud of.”, “I take a positive attitude toward myself”.

#### Demographic Questionnaire (DQ)

The DQ was designed for the current study to collect participants’ sociodemographic information, including age, gender, educational level, place of birth, family income, persons they are living with, and presence of financial problems, addictive behaviors, or chronic illness.

In addition to completing the EBS-C and DQ, the participants were asked to anonymously complete a self-administered questionnaire comprising the six measures described above (i.e., CTS2, BDI-II, GAD-7, ISEL-12, RAS, and RSES) to determine the EBS-C’s convergent validity.

### Data analyses

Construct validity assesses the degree to which a scale measures what it is supposed to measure. Three statistical methods, namely, factor analysis, known-groups validity analysis, and convergent validity analysis, were adopted in this study to determine the construct validity of the EBS-C.

Factor analysis was performed using a two-step procedure [52]. The first step was exploratory factor analysis (EFA), which was conducted to explore the scale’s factorial structure using robust maximum likelihood (MLR) estimation procedures as continuous observed variables moderately deviate from normality [53–55]. The second step was confirmatory factor analysis (CFA), a structural equation modeling approach using MLR estimation. The total sample of 718 participants was randomly split into two halves (Sample A and Sample B), each comprising 359 participants. The chi-square (χ^2^) results revealed no significant differences (p < .05) in the sociodemographic characteristics of the two samples. Sample A was used for EFA to select appropriate items and factors, with the number of factors determined by the parallel analysis and the scree plot with eigenvalue greater than 1. Sample B was used to perform CFA to confirm the structure of the scale. The Cronbach’s alphas of the EBS-C results for Samples A and B were .90 and .92, respectively, indicating a substantial degree of internal consistency [56].

Because the sensitivity of the sample size may have affected the chi-square statistics, other fit indices, including the comparative fit index (CFI), root mean square error of approximation (RMSEA), Tucker-Lewis index (TLI), and standardized root mean square residual (SRMR) were also compiled. A CFI value of .97 is a better indication of a good model fit than the more frequently used cutoff value of .95 [57, 58]. RMSEA is an indicator of model error per degrees of freedom, reflecting the average size of model misfit. RMSEA values ≤ .05 are considered indicative of a good fit, values between .05 and .08 of an adequate fit, and values between .08 and .10 of a mediocre fit, whereas values > .10 are unacceptable [59]. SRMR values < .05 indicate a good fit [57], whereas values smaller than .10 can be interpreted as acceptable.

To analyze known-groups validity, the statistical differences between different abuse histories were compared using analysis of variance (ANOVA).

To determine the scale’s convergent validity, correlation analysis was performed to examine whether the EBS-C was significantly correlated with the other measures. The magnitude of the correlation coefficient (r) determines the strength of the correlation [60].

The descriptive analyses and EFA were conducted using SPSS Version 24.0, and all CFA analyses were performed using Mplus Version 7.0. The level of significance was set at 5% (p < .05).

## Results

### Participant characteristics

The sample comprised 718 Chinese participants. The mean age of the sample was 21.4 years (SD = 2.0), with a range of 18 to 24 years. Just over half the sample (50.4%) was male; 96.6% of participants had never been married, 49.7% were students, 45.0% were employees, 57.8% had attained a degree level of education or above, and 34.7% had a monthly personal income between HK$10,000 and HK$19,999. The economic activity status was recorded according to the report of participants. Most students in Hong Kong live at home with their parents because of the expensive rent, limited accommodation provision in colleges, and the efficient public transport systems in Hong Kong. A few participants reported having financial difficulty in the past year, with 41.5% of them without monthly income. Participants’ demographic characteristics are summarized in Table 1.

**Table 1:** Demographic characteristics of the participants (*N* = 718).

	N	%
**Gender**		
**Male**	362	50.4
**Female**	356	49.6
**Age (years) [M, SD]**	21.4 (2.0)	
**Marital status**		
**Never married**	693	96.6
**Married/Cohabitating**	24	3.3
**Divorced**	1	0.1
**Economic activity status**		
**Employee**	323	45.0
**Student**	357	49.7
**Homemaker**	4	0.6
**Unemployed**	34	4.7
**Educational attainment**		
**Secondary education, diploma, or associate degree**	303	42.2
**Degree or above**	415	57.8
**Monthly personal income**		
**No income**	298	41.5
**Below HK$10,000**	120	16.7
**HK$10,000–19,999**	249	34.7
**HK$20,000 or above**	51	7.1
**Received financial support in past year**	10	1.4
**Experienced financial difficulty in past year**	8	1.1
**Experienced chronic illness in past year**	6	0.8

### EFA and internal consistency reliability

The Kaiser-Meyer-Olkin (KMO) measure was used to verify the sampling adequacy for EFA. A KMO value of .92, which is well above the acceptable limit of .50 [61], was obtained. The statistically significant result of Bartlett’s test of sphericity (χ^2^ = 1696, df = 45, p < .001) indicated that the correlations between the ten EBS-C items were sufficiently strong to perform EFA [61, 62].

EFA using MLR estimation procedures was performed for factor extraction. One factor with an eigenvalue above 1 (5.36) was found to explain 51.30% of the total variance in the ten EBS-C items. The parallel analysis based on minimum rank factor analysis revealed that one factor should be extracted, since adding more factors did not contribute to explain more variable in our data than in a random dataset. A scree plot also indicated the appropriateness of the one-factor solution, with a sharp descent in the curve from the first to second data points occurring where the eigenvalue of the second data point was below 1 (0.9). Table 2 shows the EFA factor loadings. Each of the items had a relatively high loading (ranging from .67 to .80) on the single factor, again indicating the appropriateness of the one-factor solution. The comparative fit indices of the model were above the cut-off values (CFI = .97, TLI = .96), and the model residuals were fair (SRMR = .03, RMSEA = .05). These fit indices indicated that the model fitted well the data.

**Table 2:** Exploratory factor analysis (*N* = 359).

Item	Factor 1	M	SD	Skew	Kurtosis	r_item-total_
**1 My partner makes me feel unsafe even in my own home.**	.72	1.55	0.92	1.97 (.13)	4.1 (.26)	0.67
**2 I feel ashamed of the things my partner does to me.**	.70	1.53	0.89	2.15 (.13)	5.14 (.26)	0.66
**3 I try not to rock the boat because I am afraid of what my partner might do.**	.67	1.73	1.06	1.58 (.13)	2.11 (.26)	0.64
**4 I feel like I am programmed to react a certain way to my partner.**	.63	1.68	1.00	1.55 (.13)	1.85 (.26)	0.60
**5 I feel like my partner keeps me prisoner.**	.70	1.57	0.85	1.69 (.13)	2.8 (.26)	0.66
**6 My partner makes me feel like I have no control over my life, no power, no protection.**	.77	1.51	0.84	2.22 (.13)	6.25 (.26)	0.73
**7 I hide the truth from others because I am afraid not to.**	.65	1.54	0.93	1.91 (.13)	3.17 (.26)	0.61
**8 I feel owned and controlled by my partner.**	.67	1.64	0.96	1.71 (.13)	2.87 (.26)	0.63
**9 My partner can scare me without laying a hand on me.**	.76	1.46	0.79	1.97 (.13)	4.01 (.26)	0.72
**10 My partner has a look that goes straight through me and terrifies me.**	.68	1.48	0.87	2.37 (.13)	6.6 (.26)	0.63
**KMO measure of sampling adequacy**	.92					
**Barlett’s test of sphericity**	< .001					
**χ**^**2**^ **(*df*)**	1696 (45)					
**Number of factors**	1					
**Eigenvalue**	5.36					
**Cumulative % of total variance explained**	51.30%					
**Cronbach’s alpha**	.90					

Extraction method: Robust Maximum Likelihood (MLR)

*M* = Mean; *SD* = standard deviation; r_item-total_ = item-total corrected correlation. The standard error for the skew and kurtosis statistics are presented in parenthesis.

### CFA

**Fig 1:**
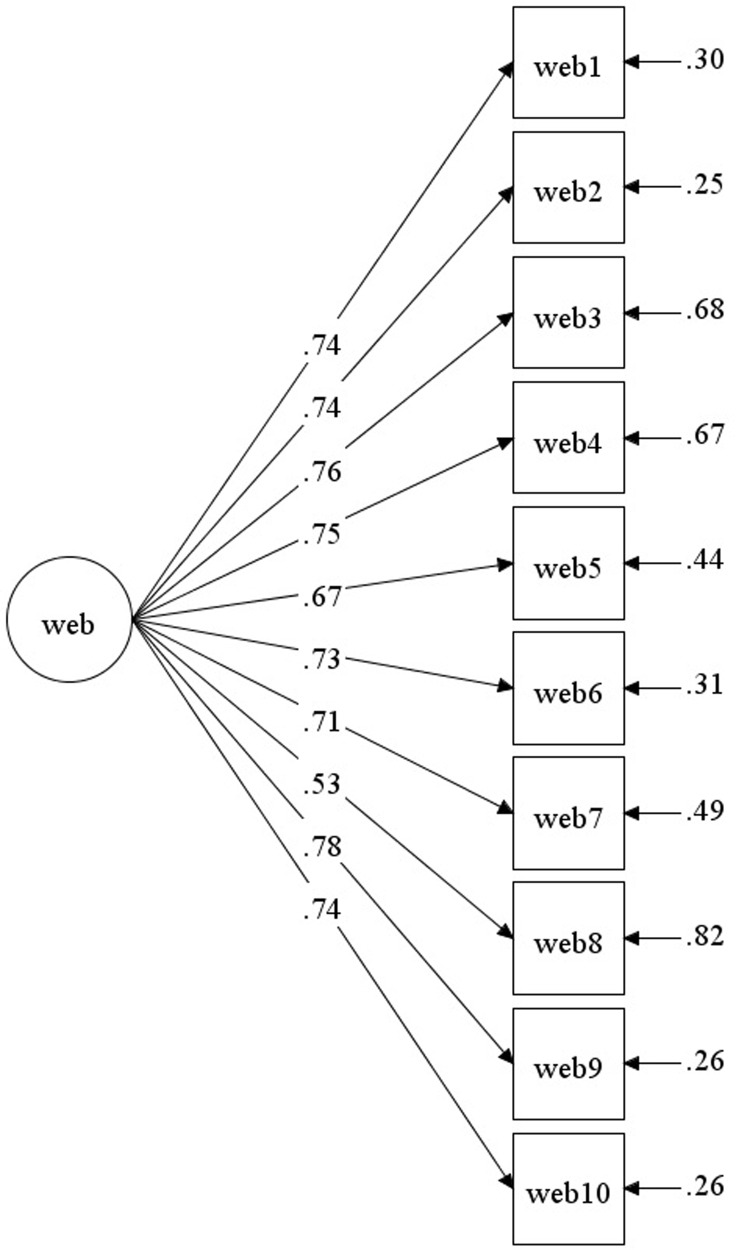
Standardized estimates of CFA model of the experience of battering scale (Chinese) (EBS-C).

Using Sample B (*N* = 359), CFA specified a one-factor model for the EBS-C. The goodness-of-fit indices of CFA and standardized estimates of the one-fact model are shown in Table 3 and Fig 1, respectively. The CFA results indicate a good model fit, with the model displaying a significant chi-square value of 69 (df = 35, p < .001). The values of the model fit measures were all above the suggested cut-off value of .95 [57, 58], with CFI equal to .97 and the TLI equal to .96. The RMSEA value was .05, lower than the alert value of .08 [63] and thus indicative of adequate fit [59]. The SRMR value was .03, indicating a good fit [57]. Hence, the one-factor model was confirmed by CFA.

**Table 3:** Goodness-of-fit indices of confirmatory factor analysis (*N* = 359).

Model	χ^2^	df	CFI	TLI	SRMR	RMSEA (95% Confidence Interval)
**One-factor model**	69	35	.97	.96	.03	.05 (.03; .07)

### Known-groups validity

The results of known-groups validity analysis are presented in Table 4. The mean EBS-C scores were found to be the highest among the participants who had experienced physical assault and injury, psychological aggression, or sexual abuse in the past year and the lowest among those in non-abusive relationships. The differences among all group combinations were found to be statistically significant for both annual prevalence (p < .001) and lifetime prevalence (p < .001).

**Table 4:** Known-groups validity (*N* = 718).

Abuse History	EBS-C			
	mean (SD)	F-test	p value	Partial Eta—squared
**Annual Prevalence** ^a^ ^b^		9.15	< .001	.026
**No abuse**	15.54 (6.54)			
**Psychological aggression only**	16.95 (5.84)			
**Physical assault/injury/psychological aggression/sexual abuse**	19.03 (9.92)			
**Lifetime Prevalence** ^a^ ^b^		12.06	< .001	.034
**No abuse**	15.28 (6.21)			
**Psychological aggression only**	16.94 (5.87)			
**Physical assault/injury/psychological aggression/sexual abuse**	18.84 (9.98)			

^a^ Significant differences for all possible group combinations.

^b^ Significant differences for all possible group combinations formed separately for male and female participants.

### Convergent validity

To assess convergent validity, correlation analysis was performed to determine whether the EBS-C was significantly correlated with the other measures (i.e., CTS2, BDI-II, GAD-7, ISEL-12, RAS, and RSES). The results showed it to be positively correlated with the CTS2 subscales of physical assault (r = .20, p < .01), injury (r = .15, p < .01), psychological aggression (r = .13, p < .01), and sexual abuse (r = .17, p < .01), as well as with depressive symptoms measured by the BDI-II (r = .15, p < .01) and generalized anxiety disorder measured by the GAD-7 (r = .17, p < .01). Furthermore, the EBS-C was negatively correlated with the level of interpersonal support measured by ISEL-12 (r = -.27, p < .01), relationship assessment measured by RAS (r = -.36, p < .01), and self-esteem measured by the RSES (r = -.22, p < .01). All of the correlations were consistent with our theoretical estimation.

Because the two known groups differed demographically, it was important to confirm the EBS-C’s association with the construct validity measures after partialling out the effects of the demographic variables (i.e., age, gender, marital status, economic activity status, educational attainment, monthly personal income, financial support in past year, financial difficulty in past year, and chronic illness in past year). This partial correlational analysis showed the EBS-C to be significantly correlated with the other measures. Partial correlation was further examined after controlling for the effects of having experienced physical assault, injury, psychological aggression, and/or sexual abuse, as well as the demographic variables. The results, which are reported in Table 5, showed the significant correlations to remain.

**Table 5:** Convergent validity: Correlations and partial correlations between EBS-C and constructed measures (*N* = 718).

Scale	Correlation	Partial Correlation ^a^	Partial Correlation ^b^
CTS2 –Physical assault	.20**	.21**	-
CTS2 –Injury	.15**	.17**	-
CTS2 –Psychological aggression	.13**	.14**	-
CTS2 –Sexual abuse	.17**	.19**	-
BDI-II	.15**	.18**	.14**
GAD-7	.17**	.21**	.19**
ISEL-12	-.27**	-.27**	-.26**
RAS	-.36**	-.34**	-.33**
RSE	-.22**	-.21**	-.21**

^a^ Controlling for demographic factors (age, gender, marital status, economic activity status, educational attainment, monthly personal income, receipt of financial support in past year, experience of financial difficulty in past year, experience of chronic illness in past year).

^b^ Controlling for demographics and CTS2 scores (physical assault, injury, psychological abuse, and sexual abuse).

** p < .01.

## Discussion

To develop and validate a culturally appropriate traditional Chinese version of the WEB scale using gender-neutral questions, Experience of Battering Scale-Chinese (EBS-C) to measure the psychological vulnerability experienced by victims of IPV, this study adopted the methods of translation and back translation, expert review, cognitive debriefing, and test-retest reliability assessment. The results indicated that the EBS-C is easy to comprehend, clear, and stable over time.

EFA using robust maximum likelihood (MLR) estimation procedures was performed. The results of the parallel analysis and a scree test of the inflexion point with an eigenvalue larger than 1 suggested a single-factor solution, which was consistent with the structure of the original WEB scale [13]. Consequently, a single-factor measurement model for the EBS-C was specified and verified via CFA. All of the model fit indices showed a good fit for the measurement model, and the EBS-C was also found to exhibit a high degree of internal consistency reliability.

According to their abuse history, the study participants were classified into three groups: (a) those in a non-abusive relationship, (b) those experiencing psychological aggression alone, and (c) those experiencing physical assault and injury, psychological aggression, or sexual abuse. The known-groups validity of the EBS-C was tested by examining whether the mean scores differed significantly among the three groups. The results indicated that participants’ abuse history could be identified by whether they received high or low EBS-C scores, thus supporting the notion that psychological vulnerability can be constructed as a continuum.

The convergent validity of the EBS-C was assessed by determining its degree of correlation with a number of measures theorized to be associated with IPV. The results of this analysis showed the EBS-C to be significantly associated with the CTS2 subscales (i.e., physical assault, injury, psychological aggression, and sexual abuse), as well as depressive symptoms, generalized anxiety disorder, interpersonal support, relationship assessment, and self-esteem [19, 42, 43], although the correlations were not strong. The associations remained significant after controlling for participants’ demographic characteristics.

The findings of this study provide clear support for the validity and reliability of the EBS-C, showing it to be a methodologically sound tool for measuring psychological vulnerability in the IPV context in the Chinese population. Further, consistent with the original WEB scale, the EBS-C addresses concerns over measuring the psychological vulnerability of victims in one basket rather than via widely used instruments that measure physical assault and psychological abuse separately [13]. Smith and her colleagues (4) pointed out that IPV victims’ behavior, views of self and their own lives were continuously affected by battering and coping experience. Therefore, battering is a process of vulnerability, loss of power and control experiences of a party caused by another party’s exercising of power using different forms of force. Thus, similar to that of WEB, EBS-C, is a measure of all possible behaviors or reactions that IPV victims have because of IPV exposure, rather than the occurrence of battering incidents and physical acts of violence. Smith’s framework of battering included psychological vulnerability [13]. Besides, the person who uses force in an intimate relationship may be an act for protection from violence as a victim rather than an abuser of IPV. The measurement of psychological vulnerability can enrich the understanding of the situation and role of an individual in IPV and clarify the confusion of mutual violence situation, an individual is a victim and perpetrator in IPV, under the measurement of the frequency of IPV-related behaviors. In addition, the association of psychological vulnerability of IPV victims to their mental health and social relationship, including depressive symptoms, generalized anxiety disorder, low self-esteem, perceived low social support and relationship satisfaction, is found. The EBS-C will not only be a useful tool to assess the victimization of IPV but also a measurement of the severity level of psychological vulnerability and its impacts on health and social life of IPV victims in the Chinese community.

Further, the findings of Houry et al. [19] support the use of the WEB scale as a gender-neutral scale for measuring the abuse of power, control, fear in an intimate relationship in both men and women. In the current study, the known-groups validity of the EBS-C was also tested on the male and female participants, with the results indicating that it can be adopted to identify psychological vulnerability in IPV victims of either sex. Our translation of the WEB scale into Chinese will allow social service or healthcare providers to obtain more detailed information from Chinese service users to evaluate the severity of the abuse they are experiencing so as to provide timely intervention and prevent IPV aggravation.

One limitation of this study was its use of convenience sampling, although participants were recruited from different sites. Generalization of the results should be treated with caution. Future research should recruit a more representative sample by adopting a random sampling method. Another limitation was the study’s cross-sectional design, meaning that its findings concern associations rather than cause-effect relationships. Finally, all of the participants were between the ages of 18 and 24. Other age groups and gender differences on psychological vulnerability in the IPV context should be investigated in future research.

## Conclusion

This study successfully developed and validated the first Chinese version of the WEB scale and showed it to have sufficient reliability and validity for use with young adults aged 18 to 24. The validated EBS-C will not only assist the helping professions and healthcare providers in identifying and assessing Chinese IPV victims to provide them with effective interventions to address their psychological vulnerability but will also facilitate cross-cultural research and cross-contextual comparisons. The study contributes to the research work on IPV in a Chinese community in order to enrich literature in the future by furthering knowledge of IPV victimization by incorporating victims’ actual experience and psychological vulnerability in a Chinese context.

## Supporting information

S1 File (DOCX)
