# Supplementary material for: Validation of the scale for assessing the psychological vulnerability and its association with health of intimate partner violence victims in Chinese young adult population
Source: PLoS One. 2020 Jul 6;15(7):e0235761. doi: 10.1371/journal.pone.0235761 (PMC7337304; doi:10.1371/journal.pone.0235761)
Supplement: S1 File — (DOCX) [file pone.0235761.s001.docx]

**關係衝突與健康調查**

你好，我係香港大學研究員，香港大學社會工作及社會行政學系，正進行一項**「關係衝突與健康」**研究，旨在了解家庭結構，衝突與暴力經歷，對健康及人際關係的影響。研究所得的資料將會作學術用途。該研究的結果會提供很多必要資訊以幫助我們制定協助相關的服務。

訪問係用不記名方式進行，所得資料會嚴加保密。非常多謝你的參與。

1. **個人狀況**

|  | **年齡 : _______________________** |
| --- | --- |
|  | **性別**  (1) □ 男  (2) □ 女 |
|  | **若主要居住地方是香港，自何時起在香港居住？**  (1) □ 出生至今  (2) □ 自______年至今 (年份，如1953年) |
|  | **你是否家庭成員同住?**  (1) □ 是  (2) □ 否，上一次見面是______個月前 |
|  | **教育程度:**   - - - - 1. □ 小學或以下         2. □ 中學，文憑或副學士         3. □ 大專或以上 |
|  | **婚姻狀況**   - - - - 1. □ 從未結婚         2. □ 已婚/同居         3. □ 離婚 |
|  | **有沒有工作或做緊生意？**   - - - - 1. □ 僱員         2. □ 學生         3. □ 料理家務者         4. □ 沒有工作 |
|  | **每月個人收入:**   - - - - 1. □ 沒有收入         2. □ 10,000以下         3. □ 10,000 – 19,999         4. □ 20,000或以上 |
|  | **有沒有長期病患?**  (1) □ 有  (2) □ 沒有 |
|  | **是否經常吸煙？**  (1) □ 有，通常一日食幾多支煙：_____________ 支  (2) □ 無，從來沒有  (3) □ 已戒 |
|  | **有無賭博？**  (1) □ 有，平均幾耐賭一次： ____________ 日  (2) □ 無，從來沒有  (3) □ 已戒 |
|  | **有無飲酒？**  (1) □ 有，平均幾耐吸毒一次： ____________ 日  (2) □ 無，從來沒有  (3) □ 已戒 |
|  | **有無吸毒 (例如服用可卡因、海洛英或鴉片等毒品) ？**  (1) □ 有，平均幾耐濫用一次： ____________ 日  (2) □ 無，從來沒有  (3) □ 已戒 |
|  | **你的家庭現時是否受到債務的困擾及/或追債的纏擾？**  (1) □ 是  (2) □ 否  (3) □ 不適用/冇意見 |

1. **自尊心量表 (RSES)**

以下是一些句子形容你對自己的感受。 如果句子很表達你的感受請在1 (很同意) 的格內填上“🗸”號，如果你只是同意句子的形容便 🗸 2 (同意) 的空格，不同意便 🗸 3 (不同意) 的空格，很不同意的話請你 🗸 4 (很不同意) 的空格。

|  | (1)  很  同  意 | (2)  同  意 | (3)  不  同  意 | (4)  很  不  同  意 |
| --- | --- | --- | --- | --- |
| 1. 整體來說，我滿意自己。 | □ | □ | □ | □ |
| 2. 有時我會覺得自己一點好處都沒有。 | □ | □ | □ | □ |
| 3. 我覺得自己有不少優點。 | □ | □ | □ | □ |
| 4. 我能夠做到與大部份人的表現一樣好。 | □ | □ | □ | □ |
| 5. 我認為自己沒有什麼可以值得自豪。 | □ | □ | □ | □ |
| 6. 有時我十分覺得自己毫無用處。 | □ | □ | □ | □ |
| 7. 我覺得自己是個有價值的人，最低限度我與其他  人有一樣的價值。 | □ | □ | □ | □ |
| 8. 我希望我能夠多一些尊重自己。 | □ | □ | □ | □ |
| 9. 從各方面看來，我是較傾向覺得自己是一個失敗者。 | □ | □ | □ | □ |
| 10. 我用正面的態度看自己。 | □ | □ | □ | □ |

1. **抑鬱自評問卷 (BDI-II)**

這份問卷共有21組的句子，每一組都有幾個選項。請你仔細閱讀每一組的句子後，從中選出一個最能夠表達你**最近兩個星期來(包括今天)** 所感受的句子，並將此選項左邊的數字圈起來。

| BD1. 悲傷   1. 我並不覺得悲傷 2. 我大部分的時間都覺得悲傷 3. 我時時刻刻都覺得悲傷 4. 我悲傷或不快樂已到我不能忍受的程度 | BD2. 悲觀   1. 我對於自己的將來並不氣餒 2. 和以往比較,我現在對自己的將來覺得沮喪 3. 我並不期望自己將來會有任何作為 4. 我覺得自己的將來沒希望,而且只會愈糟 |
| --- | --- |
| BD3. 失敗經驗   1. 我並不覺得自己是一個失敗者 2. 我遭受的失敗次數多於應有的   2. 回顧過去，我所能看到的就是許多的失敗  3. 身為一個人，我覺得自己完全失敗 | BD4. 失去樂趣   1. 對於我喜愛的事物,我和往常一樣獲得樂趣 2. 我並不如往常那般享受我喜愛的事物   2. 對於以往我喜愛的事物，我幾乎不再獲得樂趣  3. 對於以往我喜愛的事物，我已無法獲得任何樂趣 |
| BD5. 罪惡感/內咎   1. 我並不特別覺得有罪惡感(內咎) 2. 對於許多我所做或該做而沒有做到的事,我覺得有罪惡感(內咎) 3. 大部份的時間,我都覺得很有罪惡感(內咎) 4. 我時時刻刻都覺得有罪惡感 | BD6. 受懲罰感   1. 我不覺得自己正在受懲罰 2. 我覺得自己可能會受懲罰 3. 我預期自己定會受懲罰 4. 我覺得自己正在受懲罰 |
| BD7. 討厭自己   1. 我對自己的感覺仍舊和以往一樣 2. 我對自己失去了信心 3. 我對自己感到失望 4. 我討厭我自己 | BD8. 自我批評/自責   1. 我並不比平時多於批評或責怪自己 2. 我比以前更會批評自己 3. 我對自己所有的錯誤都責怪自己 4. 我對於所發生的每件壞事都會責怪自己 |

| BD9. 自殺念頭  0. 我並無任何自殺念頭  1. 我有自殺的念頭，但我不會真的去做  2. 我想去自殺  3. 如果有機會，我會真的自殺 | BD10. 哭泣  0. 我並沒有比平時哭得多  1. 我比以前較常哭泣  2. 每一件小事都會使我哭泣  3. 我很想哭泣，但哭不出來 |
| --- | --- |
| BD11. 心煩意亂  0. 和平常相比,我並不感到特別坐立不安或痛苦  1. 我覺得比平常更坐立不安或痛苦  2. 我是那樣坐立不安或心煩意亂,使我很難安定下來  3. 我是那麼坐立不安或心煩意亂,使我必須不斷地走動或做些事 | BD12. 失去興趣  0. 我並沒有對其他人或活動失去興趣  1. 我對其他人或事物的興趣比以前少一點  2. 我失去了大部份對其他人或事物自了興趣  3. 我對任何事情都提不起興趣來 |
| BD13. 優柔寡斷/猶豫不決   1. 我和往常一樣容易做決定 2. 我比平常更難做決定 3. 和以往相比,我在做決定時有極大的困難 4. 我完全無法做任何決定 | BD14. 無價值感   1. 我並不覺得自己是沒有價值的 2. 我如今不再認為自己那麼有價值 3. 我覺得自己比別人更沒有價值   3. 我覺得自己一點價值也沒有 |
| BD15. 失去精力   1. 我具有和往常一樣的精力 2. 我的精力比以前減少了 3. 我沒有足夠的精力去做很多事情   3. 我沒有足夠的精力去做任何事情 | BD16. 睡眠習慣的改變  0. 我的睡眠習慣和以前一樣，沒有任何改變  1a. 我比平常睡得多一些  1b. 我比平常睡得少一些  2a. 我比平常睡得更多  2b. 我比平常睡得更少  3a. 我差不多整天都在睡  3b. 我比以前早1-2小時醒來，而且一醒來就很難再入睡 |
| BD17. 煩躁易怒   - 1. 我不會比平常煩躁易怒   2. 我比平常較煩躁易怒   3. 我比平時更加煩躁易怒   3. 我時時刻刻都煩躁易怒 | BD18. 食慾改變  0. 我的食慾和以前一樣,並沒有任何改變  1a. 我的食慾比平常差一些  1b. 我的食慾比平常好一些  2a. 我的食慾比以前差很多  2b. 我的食慾比平常好很多  3a. 我完全沒有食慾  3b. 我時時刻刻都渴望食物 |
| BD19. 難以專注   1. 我和往常一樣能夠專注 2. 我比平常較無法專注   2. 我很難長時間專注在任何事情上  3. 我發現我不能夠專注在任何事情上 | BD20. 疲倦或疲累   1. 我和平常一樣，不覺得更疲倦或疲累 2. 我比平常容易疲倦或疲累   2. 我太疲倦或疲累，以致於許多以前常做的事都無法做  3. 我非常疲倦或疲累，以致於大部分以前常做的事都無法再做 |
| BD21. 失去對性方面的興趣   1. 我對性的興趣在最近並不覺得有任何改變 2. 我對性不再那麼感興趣 3. 我現在對性的興趣少了很多 4. 我已完全喪失對性的興趣 |  |

1. **廣泛性焦慮量表– 7  (GAD- 7)**

在**過去兩個星期中(包括今天)**，以下的情況煩擾你有多少？請選出最適用於你身上的答案。

|  | 完全  沒有  (0) | 幾天  (1) | 一半以上的天數  (2) | 幾乎每天  (3) |
| --- | --- | --- | --- | --- |
| 1. 感到緊張、不安或煩躁 | □ | □ | □ | □ |
| 1. 無法停止或控制憂慮 | □ | □ | □ | □ |
| 1. 過份憂慮不同的事情 | □ | □ | □ | □ |
| 1. 難於放鬆 | □ | □ | □ | □ |
| 1. 心緒不寧以至坐立不安 | □ | □ | □ | □ |
| 1. 容易心煩或易怒 | □ | □ | □ | □ |
| 1. 感到害怕，就像要發生可怕的事情 | □ | □ | □ | □ |

1. **人際支持評估表 (ISEL) — 一般人口**

本評估表由一系列陳述組成，各項陳述可能會適用於你身上。如果你確定有關陳述適用於你身上，請選擇「絕對是」一欄，如果你認為有關陳述適用於你身上但不能完全肯定，請選擇「可能是」一欄。同樣，如果你確定有關陳述不適用於你身上，請選擇「絕對不是」一欄，如果你認為有關陳述不適用於你身上但不能完全肯定，請選擇「可能不是」一欄。

|  | 絕對不是 | 可能不是 | 可能是 | 絕對是 |
| --- | --- | --- | --- | --- |
| 1. 如果我想去旅行一日（例如，上山、去沙灘或郊外），會很難找人陪我。 | 0 | 1 | 2 | 3 |
| 1. 我覺得沒有人能夠分享我最私人的擔憂與恐懼。 | 0 | 1 | 2 | 3 |
| 1. 如果我病了，會很容易找到人幫我處理我的日常家務。 | 0 | 1 | 2 | 3 |
| 1. 我可以向某人尋求有關處理我的家庭問題的意見。 | 0 | 1 | 2 | 3 |
| 1. 如果我在某個下午決定在傍晚時看電影，會很容易找到人陪我。 | 0 | 1 | 2 | 3 |
| 1. 當我需要建議如何處理私人難題時，我知道我可以向某人求助。 | 0 | 1 | 2 | 3 |
| 1. 我不常獲邀與其他人一起活動。 | 0 | 1 | 2 | 3 |
| 1. 如果我需要離開本市幾星期，會很難找人替我打理房屋（植物、寵物、花園等）。 | 0 | 1 | 2 | 3 |
| 1. 如果我要和別人共進午餐，會很容易找到人陪我。 | 0 | 1 | 2 | 3 |
| 1. 如果我在遠離居所後走失，可以致電某人來找我。 | 0 | 1 | 2 | 3 |
| 1. 如果家中出現危機，會很難找人給我良好意見來處理問題。 | 0 | 1 | 2 | 3 |
| 1. 如果我搬新屋需要幫助，會很難找人幫我。 | 0 | 1 | 2 | 3 |

1. **關係衝突行為**

不論親密伴侶(例如男/女朋友)之間相處得如何融洽，有時候也會意見不合、惱怒對方、彼此有不同的要求，或因

心情欠佳、疲倦或其他原因而爭吵或打架。配偶之間會用不同的方法去處理衝突，以下列舉的項目是當彼此有意

見不合時，有可能會發生的事情。

**請以目前或最近期的親密伴侶為對象，回答以下每條題目，並請固定以這位親密伴侶為回答問題的參照。**

**注意：填寫次數時只須憑印象，選擇最接近的類別即可。**

**1. 關於口角方面：**

|  |  | 親密伴侶曾對我作過下列行為 | | | | | | | | | |
| --- | --- | --- | --- | --- | --- | --- | --- | --- | --- | --- | --- |
|  |  | 從來沒有發生過 | 過去十二個月發生的次數 | | | | | | 十二個月以前曾經發生 | 六個月內曾經發生 | 拒絕回答 |
|  |  |  | 1次 | 2次 | 3-5次 | 6- 10次 | 11-  20次 | 20次以上 |  |  |  |
|  |  | (0) | (1) | (2) | (3) | (4) | (5) | (6) | (7) | (8) | (9) |
| a | 侮辱或咒罵對方 | □ | □ | □ | □ | □ | □ | □ | □ | □ | □ |
| b | 曾向對方喊叫或呼喝 | □ | □ | □ | □ | □ | □ | □ | □ | □ | □ |
| c | 意見不合時，跺腳重踏或撞門離開 | □ | □ | □ | □ | □ | □ | □ | □ | □ | □ |
| d | 曾講一些刁難  對方嘅說話 | □ | □ | □ | □ | □ | □ | □ | □ | □ | □ |
| e | 曾用難聽嘅說話，  話對方肥或醜 | □ | □ | □ | □ | □ | □ | □ | □ | □ | □ |
| f | 曾破壞屬於對方嘅物件 | □ | □ | □ | □ | □ | □ | □ | □ | □ | □ |
| g | 曾指責對方是一個  卑劣討厭的配偶 | □ | □ | □ | □ | □ | □ | □ | □ | □ | □ |
| h | 曾威嚇要毆打或  搵野掟對方 | □ | □ | □ | □ | □ | □ | □ | □ | □ | □ |
| i | 曾恐嚇會傷害  對方嘅家人 | □ | □ | □ | □ | □ | □ | □ | □ | □ | □ |
| j | 曾經話過想死 | □ | □ | □ | □ | □ | □ | □ | □ | □ | □ |
| k | 曾經話過要攬住一齊死 | □ | □ | □ | □ | □ | □ | □ | □ | □ | □ |
| l | 意見不和時會不瞅不睬 | □ | □ | □ | □ | □ | □ | □ | □ | □ | □ |
| m | 曾恐嚇會傷害孩子 | □ | □ | □ | □ | □ | □ | □ | □ | □ | □ |

**2. 關於使用武力方面：**

|  |  | 親密伴侶曾對我作過下列行為 | | | | | | | | | |
| --- | --- | --- | --- | --- | --- | --- | --- | --- | --- | --- | --- |
|  |  | 從來沒有發生過 | 過去十二個月發生的次數 | | | | | | 十二個月以前曾經發生 | 六個月內曾經發生 | 拒絕回答 |
|  |  |  | 1次 | 2次 | 3-5次 | 6- 10次 | 11-  20次 | 20次以上 |  |  |  |
|  |  | (0) | (1) | (2) | (3) | (4) | (5) | (6) | (7) | (8) | (9) |
| a | 搵野掟對方，而可能會整傷對方 | □ | □ | □ | □ | □ | □ | □ | □ | □ | □ |
| b | 曾扭對方嘅手臂或 扯對方嘅頭髮 | □ | □ | □ | □ | □ | □ | □ | □ | □ | □ |
| c | 曾推撞或推開對方 | □ | □ | □ | □ | □ | □ | □ | □ | □ | □ |
| d | 曾抓住對方 | □ | □ | □ | □ | □ | □ | □ | □ | □ | □ |
| e | 曾掌摑對方 | □ | □ | □ | □ | □ | □ | □ | □ | □ | □ |
| f | 曾用刀或利器 指向對方 | □ | □ | □ | □ | □ | □ | □ | □ | □ | □ |
| g | 曾用拳頭或搵野打對方，可能會整傷對方 | □ | □ | □ | □ | □ | □ | □ | □ | □ | □ |
| h | 曾勒住對方嘅頸 | □ | □ | □ | □ | □ | □ | □ | □ | □ | □ |
| i | 曾把對方大力撞向牆壁 | □ | □ | □ | □ | □ | □ | □ | □ | □ | □ |
| j | 曾經毆打對方 | □ | □ | □ | □ | □ | □ | □ | □ | □ | □ |
| k | 曾故意燒傷或燙傷對方 | □ | □ | □ | □ | □ | □ | □ | □ | □ | □ |
| l | 曾經踢對方 | □ | □ | □ | □ | □ | □ | □ | □ | □ | □ |

**3. 因使用武力而導致受傷：**

|  |  | 親密伴侶曾對我作過下列行為 | | | | | | | | | |
| --- | --- | --- | --- | --- | --- | --- | --- | --- | --- | --- | --- |
|  |  | 從來沒有發生過 | 過去十二個月發生的次數 | | | | | | 十二個月以前曾經發生 | 六個月內曾經發生 | 拒絕回答 |
|  |  |  | 1次 | 2次 | 3-5次 | 6- 10次 | 11-  20次 | 20次以上 |  |  |  |
|  |  | (0) | (1) | (2) | (3) | (4) | (5) | (6) | (7) | (8) | (9) |
| a | 曾因雙方打架/爭執而扭傷、碰瘀或割傷 | □ | □ | □ | □ | □ | □ | □ | □ | □ | □ |
| b | 曾因同對方打架，令身體痛楚，  直至第二日仍然痛 | □ | □ | □ | □ | □ | □ | □ | □ | □ | □ |
| c | 曾被對方擊中頭部而失去知覺 | □ | □ | □ | □ | □ | □ | □ | □ | □ | □ |
| d | 曾因同對方打架而求醫 | □ | □ | □ | □ | □ | □ | □ | □ | □ | □ |
| e | 曾因同對方打架本來需要求醫，但最終沒有去 | □ | □ | □ | □ | □ | □ | □ | □ | □ | □ |
| f | 曾因同對方打架/爭執而骨折 | □ | □ | □ | □ | □ | □ | □ | □ | □ | □ |

**4. 性行為方面：**

|  |  | 親密伴侶曾對我作過下列行為 | | | | | | | | | |
| --- | --- | --- | --- | --- | --- | --- | --- | --- | --- | --- | --- |
|  |  | 從來沒有發生過 | 過去十二個月發生的次數 | | | | | | 十二個月以前曾經發生 | 六個月內曾經發生 | 拒絕回答 |
|  |  |  | 1次 | 2次 | 3-5次 | 6- 10次 | 11-  20次 | 20次以上 |  |  |  |
|  |  | (0) | (1) | (2) | (3) | (4) | (5) | (6) | (7) | (8) | (9) |
| a | 做愛時，無理會對方使用安全套嘅要求 | □ | □ | □ | □ | □ | □ | □ | □ | □ | □ |
| b | 就算對方唔同意，仍堅持要同對方做愛，但無使用武力 | □ | □ | □ | □ | □ | □ | □ | □ | □ | □ |
| c | 就算對方唔同意，仍堅持要同對方口交或肛交，但無使用武力 | □ | □ | □ | □ | □ | □ | □ | □ | □ | □ |
| d | 曾以武力（例如打、按住、或使用武器）來迫對方同自己口交或肛交 | □ | □ | □ | □ | □ | □ | □ | □ | □ | □ |
| e | 曾以武力（例如打、按住、或使用武器）來迫對方同自己做愛 | □ | □ | □ | □ | □ | □ | □ | □ | □ | □ |
| f | 曾威嚇迫對方同自己口交或肛交 | □ | □ | □ | □ | □ | □ | □ | □ | □ | □ |
| g | 曾威嚇迫對方同自己做愛 | □ | □ | □ | □ | □ | □ | □ | □ | □ | □ |

1. **關係滿意度量表 (RAS)**

此部份在於瞭解您對親密關係的滿意度。請選擇最符合你情況的答案。請以現時〔或最近期〕伴侶的情況作答。

|  |  | 低 |  | 中等 |  | 高 |
| --- | --- | --- | --- | --- | --- | --- |
| 1. | 你的伴侶〔或前伴侶〕滿足你各項需求的程度如何？ | 1 | 2 | 3 | 4 | 5 |
|  |  | 非常不滿意 |  | 普通 |  | 非常滿意 |
| 2 | 整體而言，你滿意你們的關係嗎？ | 1 | 2 | 3 | 4 | 5 |
|  |  | 非常不好 |  | 尚可 |  | 非常好 |
| 3 | 和大多數人相比，你們的關係如？ | 1 | 2 | 3 | 4 | 5 |
|  |  | 從沒有想過 |  | 偶爾 |  | 時常這樣想 |
| 4 | 你是否曾想過，若不與他／她有這段關係那該多好？ | 1 | 2 | 3 | 4 | 5 |
|  |  | 非常不符合 |  | 普通 |  | 非常符合 |
| 5 | 你們的關係是否符合您原先的期待？ | 1 | 2 | 3 | 4 | 5 |
|  |  | 一點也不愛 |  | 普通 |  | 非常的愛 |
| 6 | 你有多愛他／她？ | 1 | 2 | 3 | 4 | 5 |
|  |  | 非常的少 |  | 不多不少 |  | 非常多 |
| 7 | 你們的關係存在著多少問題？ | 1 | 2 | 3 | 4 | 5 |

1. **受虐經驗量表 (EBS-C)**

以下10個句子是用來描述自己與伴侶的生活，請閱讀每個句子並圈出你有多同意或不同意該項描述。請以現時〔或最近期〕伴侶的情況作答。

|  | 非常  同意 | 部分同意 | 有點同意 | 有點不同意 | 部分不同意 | 非常不同意 |
| --- | --- | --- | --- | --- | --- | --- |
| 1. 即使在自己的家中，我的伴侶也會使我感到不安全。 | 1 | 2 | 3 | 4 | 5 | 6 |
| 1. 我對我的伴侶對我做的事感到羞恥。 | 1 | 2 | 3 | 4 | 5 | 6 |
| 1. 我盡量不破壞現狀，生怕我的伴侶會做甚麼事。 | 1 | 2 | 3 | 4 | 5 | 6 |
| 1. 我覺得自己已被預設以一定的方式去回應我的伴侶。 | 1 | 2 | 3 | 4 | 5 | 6 |
| 1. 我感到我的伴侶待我像囚犯。 | 1 | 2 | 3 | 4 | 5 | 6 |
| 1. 他/她令我感到我對自己的生活沒有控制權，自己沒有能力和保障。 | 1 | 2 | 3 | 4 | 5 | 6 |
| 1. 我要對其他人隱瞞真相，因為我害怕。 | 1 | 2 | 3 | 4 | 5 | 6 |
| 1. 我感到被我的伴侶佔有和操控。 | 1 | 2 | 3 | 4 | 5 | 6 |
| 1. 即使我的伴侶不動手傷害我，也能把我嚇倒。 | 1 | 2 | 3 | 4 | 5 | 6 |
| 1. 我的伴侶一臉看穿我的樣子，使我害怕。 | 1 | 2 | 3 | 4 | 5 | 6 |

|  |
| --- |
|  |

**問卷完成**
